# Increased Gamma Brainwave Amplitude Compared to Control in Three Different Meditation Traditions

**DOI:** 10.1371/journal.pone.0170647

**Published:** 2017-01-24

**Authors:** Claire Braboszcz, B. Rael Cahn, Jonathan Levy, Manuel Fernandez, Arnaud Delorme

**Affiliations:** 1 Université de Toulouse, UPS, Centre de Recherche Cerveau et Cognition, Toulouse, France; 2 CerCo, CNRS UMR5549, Toulouse, France; 3 University of Southern California Department of Psychiatry, Los Angeles, California, United States of America; 4 University of Southern California Brain and Creativity Institute, Los Angeles, California, United States of America; 5 Meditation Research Institute, Swami Rama Sadhaka Grama, Rishikesh, India; 6 Swartz Center for Computational Neuroscience, University of California San Diego, La Jolla, California, United States of America; University of Pécs Medical School, HUNGARY

## Abstract

Despite decades of research, effects of different types of meditation on electroencephalographic (EEG) activity are still being defined. We compared practitioners of three different meditation traditions (Vipassana, Himalayan Yoga and Isha Shoonya) with a control group during a meditative and instructed mind-wandering (IMW) block. All meditators showed higher parieto-occipital 60–110 Hz gamma amplitude than control subjects as a trait effect observed during meditation and when considering meditation and IMW periods together. Moreover, this gamma power was positively correlated with participants meditation experience. Independent component analysis was used to show that gamma activity did not originate in eye or muscle artifacts. In addition, we observed higher 7–11 Hz alpha activity in the Vipassana group compared to all the other groups during both meditation and instructed mind wandering and lower 10–11 Hz activity in the Himalayan yoga group during meditation only. We showed that meditation practice is correlated to changes in the EEG gamma frequency range that are common to a variety of meditation practices.

## Introduction

Scientific publications on meditation have dramatically increased over the last decade [1]. The recent interest in these ancient mental practices is concomitant with the development of new brain imaging technologies as well as the incorporation of mindfulness, one of the core psychological components of these practices, into psychotherapeutic and clinical interventions [2, 3]. Together these studies have demonstrated the beneficial effects of meditative practices on perception [4, 5], cognition [6, 7], emotional processing [8, 9], and neuroplasticity [10–12]. A recent meta-analysis of neuroimaging studies over about 300 meditation practitioners has shown that meditation practice is consistently associated with changes in morphology of the prefrontal cortex and body awareness regions [13]. Such changes might have an impact on the brain functioning, however fMRI-based studies can not capture the real-time dynamic of the brain activity as can be done using electroencephalography (EEG), the recording of electrical currents measurable from the surface of the scalp.

**Fig 1:**
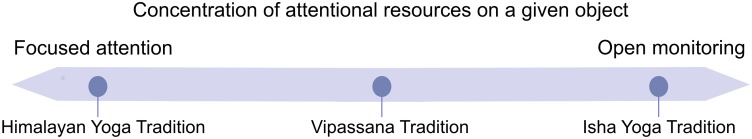
The continuum of attentional engagement, from the high level of focused attention to more diffuse“open monitoring” or “open awareness” meditation. The three meditation traditions we chose to include in our study can be placed along this continuum, each corresponding to a different focus of attention. Himalayan Yoga tradition uses a mantra to maintain the attentional focus, Vipassana tradition is primarily an open monitoring practice but the specific form assayed (as taught by S. N. Goenka) incorporates loose focus on the somatosensory awareness aspect and the Shoonya practice as taught in the Isha Yoga tradition is an open awareness meditation practice with no specific object to focus on.

Although EEG is one of the primary neuroimaging methods used to study meditation, to date no consensus has emerged on the basic effects of meditation on EEG activity. Meditative practices are usually considered to fall somewhere along a continuum of two broader categories, concentration meditations and mindfulness meditations, also referred to as “focused attention” and “open monitoring” states of attentional engagement [14] (Fig 1). Given the large diversity of meditative practices, a caveat of the early meditation studies was to involve heterogeneous groups of meditators without the appropriate control group and/or condition that are needed to assess meditation trait and state related effects [15].

The objective of this study is to examine first whether there is a consistent difference between the EEG activity of meditation practitioners compared to meditation-naive participants, and second whether different meditation practices can be distinguished based on either their state or trait effects on the EEG signal.

Most EEG studies of meditation report trait and/or state increases or decrease of power in the lower frequencies bands such as theta and alpha without clear differences between different meditation practices while studies directly assessing the EEG correlates of different practices are rare (see [15]). In addition to these changes in low frequency bands, more recent studies have found higher frequency gamma activation (>30 Hz) specifically associated with meditation state or trait effects in various meditation practices: Lutz et al. [16] found increased gamma over frontolateral and posterior electrodes in non-referential, objectless compassion meditation, Cahn et al. [17] have described increased gamma activity over parieto-occipital electrodes during open-monitoring Vipassana meditation that focuses on somatic sensation and Berkovich-Ohana et al. [18] reported increases in gamma power over posterior electrodes during an another type of open-monitoring meditation, mindfulness meditation. Higher gamma activity in experienced meditators have also been reported over parieto-occipital electrodes during periods of NREM sleep and positively correlated with the length of lifetime meditation practice [19]. In addition, Hauswald et al. [20] found that in Zen meditators scores on a mindfulness scale correlate with gamma power during meditation at frequencies above 100 Hz.

Interestingly, the EEG gamma frequency has been linked to diverse cognitive functioning, including a general neural correlate of the ongoing stream and contents of consciousness [21, 22], long-range neuronal communication underlying the “binding problem” [23, 24], visual representation [25, 26] and attention [27, 28], although this role of gamma band in perception and cognition remains controversial [29, 30]. Several studies have found correlations between increases in gamma power as the most plausible electrophysiological correlate to increases in blood supply as measured by the fMRI BOLD signal [31, 32].

The meditation traditions included in the present study each put a different emphasis on the focus of the mind during meditative practice [15, 33, 34] (see Fig 1). We chose to focus on three different meditation traditions that are representative of the main meditation styles practiced around the world to allow our results to be generalisable and comparable to findings in existing and future studies on respectively similar meditation techniques.

We included a focused meditation on mantra repetition (Himalayan Yoga Tradition; HYT), an open monitoring meditation focusing on body sensations (Vipassana; VIP) and an open awareness meditation (“Shoonya” meditation, a practice part of Isha Yoga tradition; Isha Shoonya Yoga—ISY), which is somewhat comparable to Zen Shikantaza. Both Shoonya in Sanskrit and Shikantaza in Japanese are translated as variations on the term “nothingness/doing nothing”, which is the explicit attentional goal of this practice in the Isha Yoga tradition.

Meditators were recruited based on their expertise within their specific traditions. A control group (CTR) with no prior meditation experience was also recruited. Of the three meditation practices we analyzed in this study, we can note that only studies of the Vipassana tradition have been regularly reported in the neuroimaging literature. The Himalayan yoga tradition and Isha yoga tradition while being close to other studied meditation practices (mantra meditation and zen meditation respectively) have never been directly assessed using neuroimagery.

In light of previous research by Cahn et al. [17, 35], we incorporated an instructed mind-wandering (IMW) task as a control mental state during which participants were instructed to consistently remember non-emotional autobiographical memories. This was preferred to just using resting state instructions to prevent meditators from slipping into a meditative state while at rest (a common phenomenon reported by many practitioners).

Informal discussion with pilot participants highlighted the need for a preparatory period before starting with the formal meditation practice. Thus to ensure the quality of the meditation task per se, the meditation block started with a 10 min period of breath-focus awareness to give an opportunity for meditators to get ready for their meditation practices.

Granted the growing understanding that meditative practices involve the active engagement of attentional processes and that EEG gamma activity is related to enhanced attention and neural activation, and considering the previous findings on gamma activity and meditation exposed above, we hypothesized that state effect increases in gamma power during meditation compared to the control IMW task might be a common feature of all meditation traditions and that increase in gamma power would be found as a trait effect when comparing expert meditators with naive participants. In addition, based on the reviewed literature we expected to find an effect mainly over parieto-occipital electrodes location [17–19]. We also anticipated to find meditation related differences in lower frequency bands as meditation effects on the theta and alpha frequency bands have been regularly reported in the literature [36–39]. Finally, we counted upon the breath awareness preparatory period for giving us a baseline meditative state upon which we could contrast each of the traditions specific meditation practice to investigate further any specific state effect of a particular practice.

## Materials and Methods

### Participants

Data collection took place at the Meditation Research Institute (MRI) in Rishikesh, India. Subjects which lived close to the Institute received about 10 US dollars (500 Rupees) as compensation and subjects who traveled longer distances received about 20 US dollars (1000 Rupees) in addition to reimbursement of their travel fees, accommodation and meals. About one third of the subjects (mostly from the ISY group) traveled more than 2000 km to participate in the experiment. Participants all provided written consent to participate in the study. The project was approved by the local MRI Indian ethical committee and the ethical committee of the University of California San Diego (IRB project # 090731). All meditators were chosen for inclusion in the study based on age, gender and years of practice of meditation. Control subjects were chosen for inclusion in this study based on age and gender and their absence of a meditation practice. We collected data on 20 meditators from the Vipassana tradition, 27 meditators from Himalayan Yoga Tradition, 20 meditators from Isha Shoonya Yoga tradition and 32 controls. All meditators were used to practice meditation with their eyes closed.

In the current study, we aimed at analyzing homogeneous groups of subjects and groups were formed by matching subjects based on gender and age. Following this matching procedure we obtained 4 groups of 16 subjects each. Because the Isha shoonya and Vipassana groups did not contain as many subjects as the control and Himalayan yoga groups, the matching process implied leaving out of the present analysis some control and Himalayan yoga participants. The control and the Vipassana groups contained 5 female participants, and the remaining groups 2 female participants. In each groups, 8 of the subjects performed the mind-wandering task first and 8 subjects performed the meditation task first. Table 1 indicates the statistics for the different traditions. Hours of meditation in life were estimated based on subjects reports of their daily practice and participation to full time retreat. Number of hours spent meditating during full time retreat was estimated by the subjects.

**Table 1:** Subject groups mean age and estimated hours of life-time meditation experience. Number in parentheses indicate minimum and maximum hours of meditation. Symbol ± indicates the standard deviation.

	CTR	HYT	ISY	VIP
Age	45 ± 10	49 ± 13	40 ± 10	47 ± 15
Hours of meditation	0	15475 ± 12748	2625 ± 1868	9201 ± 7759
Gender (females/16)	5	2	2	5

### Meditation practice

#### Vipassana meditation

The term Vipassana can describe diverse meditative practices [40]. All our Vipassana meditators practiced the Vipassana meditation technique as taught by S.N. Goenka [41]. The main practice of Vipassana consists in mentally scanning one by one each body part and feeling the sensations in each of these body parts. Practitioners are instructed to move their attention down from the top of the head to the tips of the toes and then in the opposite direction in a repetitive pattern, paying attention to somatic sensations. The instruction for the subject is to keep his/her attention moving and to observe, objectively and with equanimity, the sensations he/she experiences. Given the explicit focus on somatosensory sensations, this particular tradition of Vipassana is a good example of meditation practice where focused attention and open monitoring are combined. Meditators were chosen for inclusion in the study based on age, gender and years of practice of the Vipassana meditation.

#### Himalayan Yoga tradition

The Himalayan Yoga tradition is an ancient tradition consisting of many steps and paths that are integrated and interrelated with each other [42, 43]. For example: (1) deep relaxation techniques performed in “corpse position” leading to Yoga Nidra (the sleep of the Yogi); (2) maintaining correct position of the spine in sitting for meditation and practicing diaphragmatic breathing; (3) practicing breath awareness practices; (4) mentally repeating a sound or series of sounds (mantra) and at the same time focusing on the breath and, in more advanced practitioners, specific body energy centers (chakras). The mantra may also be used only as a thought without breath awareness. In this tradition there is also an emphasis made on the breath that should be flowing without pauses. For this specific experiment, Himalayan Yoga meditators mentally repeated their Mantra with or without awareness of the breath. Meditators were chosen for inclusion in this study based on age, gender and years of practice of the Himalayan Yoga meditation tradition.

#### Isha Yoga tradition

Isha Yoga meditation is a form of yoga, which includes Asanas (yoga postures), Kriyas (specific breathing techniques and body postures) and sitting meditation (Shoonya and Samyama meditations). During the Asanas and Kriyas, practitioners focus on the breath and body sensations; during the meditations, the practitioners maintain awareness of their thought process. For the purpose of the experiment, Isha meditators were asked to practice Shoonya meditation. In this meditation, the meditator goes through a process of conscious “non-doing” that purportedly creates a distance between one’s self and one’s body and mind. For the duration of the meditation, the practitioner consciously does not respond to any internal or external stimuli. Meditators were chosen for inclusion in this study based on age and gender and years of practice of the Isha Yoga meditation.

#### Control subjects

Control subjects did not have a meditation practice, although a few subjects chanted prayers as part as their daily religious rituals. Subjects for which this chanting included a meditation component were excluded. During the meditation condition, control subjects were instructed to remain aware of their breath for the duration of the recording period. The exact instruction given to them was to “keep paying attention to the sensations of the breath, both of the inhalation and the exhalation. If your mind starts to wander, please bring it back to your breath.” They were allowed to ask questions about the practice and performed the task for 5 minutes to ensure understanding of the instructions prior to starting the experiment. Control subjects were chosen for inclusion in this study based on age and gender and their absence of meditation practice.

### Procedure

Participants sat either on a blanket on the floor or on a chair for both experimental periods depending on their personal preference. They were asked to keep their eyes closed and all lighting in the room was turned off during data collection. An intercom allowed communication between the experimental and the recording room.

**Fig 2:**
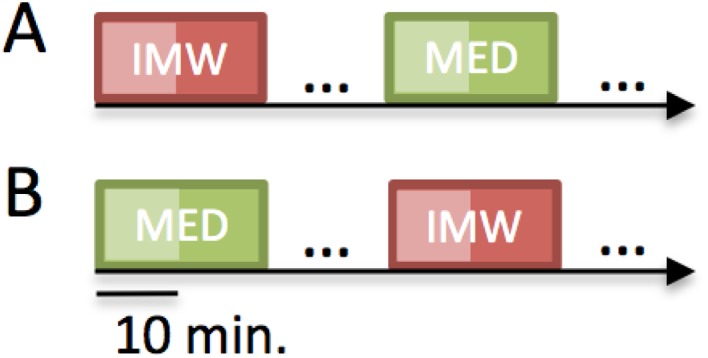
Experimental protocol. The first 10 minutes of the meditation block (MED) are considered a preparatory period helping to relax for the meditation practice. Meditators then switched to their specific meditation practice for the last 10 minutes of the block. Control subjects practiced breath watching throughout the whole MED block. For the sake of consistency, we kept the length of the instructed mind-wandering block (IMW) equal to the length of the meditation block. When analyzing the data we compared the last 10 minutes of the MED block to the last 10 minutes of the IMW block. Half the subjects passed the blocks in the order presented on panel A, the other half passed the blocks as shown on panel B.

Participants performed two sessions of 20 minutes, one of “Meditation” (MED) and the other one of “Instructed Mind Wandering” (IMW). In the MED block, participants were first instructed to pay attention to their breathing as to prepare for their meditation practice (breath focus): they were told to focus on noticing the air flowing in and out their noses, or if this was too difficult for them, they were given the alternative of attending to the sensations of their abdomen associated with inhalation and exhalation. This task was chosen as it is a basic practice given to novice beginners in meditation and common to all three meditation traditions investigated here. It was thus helping the meditators to relax and to prepare for their meditation practice. After 10 minutes participants were told—through the intercom—to practice their regular meditation practices for the remaining 10 minutes (MED; see Fig 2). In the instructed mind-wandering blocks, participants were instructed to remember autobiographical events from childhood to the most recent past. They were given a list of potential events to remember before the recording session as examples (the list involved daily childhood life, travels, etc.). They were explicitly told to avoid remembering emotionally charged events. To keep the IMW condition as close as possible to the MED condition, after the first 10 minutes participants were told—through the intercom—to continue doing the instructed mind wandering task for the next 10 minutes. In addition, after the instructed mind-wandering and meditation blocks, participants were presented with a diverse set of auditory and visual tasks—the results of which will not be reported here.

Immediately after each session, participants completed a questionnaire to assess the subjective characteristics of their mental state during the session (see below Psychometric data collection) and were allowed to stand up, stretch and walk around. The order of the sessions was counterbalanced between participants to prevent any order effects. The day preceding the study, participants were invited to visit the experimental room to practice the tasks for 10 minutes (5 minutes instructed mind-wandering and 5 minutes meditation). Participants entering the study as meditators were asked to avoid meditating before coming to the laboratory on the day of the experiment.

### Data collection

We recorded data using an 64 + 8 channels Biosemi Active-Two amplifier system and a 10–20 Headcap standard 64-channel cap from the same company. Using external electrodes, we also recorded right and left mastoid electrodes as well as vertical and horizontal electrooculogram (EOG) by placing two periocular electrodes above and below the left eye and two electrodes at both the left and right outer canthi.

The experimental room was soundproof and the floor was electrically shielded and grounded. In order to ensure good quality of EEG signal, participants were asked to wash their hair before attending the recording session and, for non scalp electrodes, their skin was carefully cleaned using an alcohol solution. All electrodes were kept within 50mV offset of the BIOSEMI system metric for measuring impedance.

### Data processing and artifact rejection

Data processing was done using the EEGLAB open source software version 12 [44] running on Matlab R2009b (The Matworks, Inc.) under a Linux operating system (Ubuntu 12.04). EEG data were first referenced to the right mastoid and down-sampled from 1024 Hz to 256 Hz. We then applied a high-pass filter at 1 Hz using an infinite impulse response (IIR) filter with a transition bandwidth of 0.3 Hz and an order of 6.

We automatically removed portions of the signal presenting non-stereotyped artifacts using the *pop_rejcont* function of the EEGLAB software [44]. The data were first segmented in 1-second epochs with 0.5-second overlap. Segments of 8 contiguous epochs in which the 0–10 Hz frequency band and the 35–128 Hz frequency band had an amplitude higher than 17 and 14 decibels respectively were labelled as artifactual. We used this rejection procedure to ensure that artifact rejection was uniform for all subjects. Rejection of low-frequency segments helped remove signals related to subjects’ head and body movements. Rejection of high frequency activity helped reject data portions of muscular activity. The data was then checked visually for any potential remaining artifact—leading in some rare cases to adjust decibels thresholds from 1 to 5 dB and rerunning the rejection procedure. We then manually identified and removed bad electrodes (from 0 to 18 bad electrodes per subject, average of 5 electrodes removed per subject). Finally, we used Infomax Independent Component Analysis (Infomax ICA) on the pruned data to reject eye movement related and muscle artifacts. For each subject, we visually identified and subtracted one to five well-characterized ICA components from the data. These components accounted for eye artifacts and temporal muscle noise. Experimenter CB used visual inspection of component scalp topographies and power spectrum to reject these artifactual ICA components [45]. To allow analysis of the ICA components at the group level, we used the EEGLAB Corrmap plugin [46] to form clusters of independent components based on the correlations of their scalps topographies.

For each subject and each of the 2 conditions meditation practice (MED) and instructed mind wandering (IMW), we applied a spectral decomposition to the continuous data of each channel. We first segmented data into 1-second long epochs with no overlap, and then performed a Fourier transformation on these epochs after tappering the signal with a Hanning window. Finally, spectral power—equal to the square of Fast Fourier Transform (FFT) amplitude—was visualized in log scale (10 times the base-10 logarithm of the spectral power).

### Psychometric data collection

Subjective psychometric data were collected both prior to the experiment and at the end of each block. During the experiment subjects were allowed to sit at a table at the end of each block and asked to complete a questionnaire regarding their state of mind during the preceding block. We used 10-points scale to assess depth of the meditation (1: not deep at all, 10: deepest) and thought density (1: lot of space between occurrence of thoughts, 10: thoughts were frequent and overlapping). In addition, subjects had to rate on a 1 to 4 scale (1: not all, 4:very much) relaxation and hindrance items extracted from the Meditation Depth Questionnaire (MEDEQ, [47]), a validated questionnaire to assess the depth dimension of meditation. The full MEDEQ consists in five categories of questions, however we only used here a subset of two categories. Relaxation items assess the feeling of ease (ie.“I felt well”, “I felt I became more patient and calm”) while hindrance items are thought to assess (emotional) blocks or difficulties that could have been encountered during the preceding period (ie. “I was bored”, “I felt drowsy and sleepy”). The same questionnaire was given prior to the experiment to assess the habitual quality of participants meditative state.

### Statistics

Analysis of variance (ANOVA) was first used to assess significance of the EEG spectral power across groups and conditions using either one-way Welch’s ANOVA [48] or mixed design ANOVA when comparing between and within subjects variables in the same model. To allow for a finer localization of the effects over electrodes, post-hoc tests were performed within EEGLAB as described below.

Statistics for EEG data were performed on the log-transformed spectral power data across channels using the permutation tests implemented in the Fieldtrip toolbox [49]. A total of 2 000 re-samples of the original data was used to assess significance. When conditions were compared within a group, permutations of paired t-test were used and when comparisons were made in between 2 groups permutations of non-paired t-tests were used. Two-tailed statistics were used by default. Correction for multiple comparisons at the electrode level was performed using the cluster-based statistics implemented in Fieldtrip [50]—using the algorithm triangulation method for finding channel neighbors and the default maxsum method for finding clusters with a cluster-forming threshold of p = 0.05. Note that the cluster correction method forces to choose a statistical threshold and does not currently allow to report individual p-value for each electrodes.

During both the instructed mind wandering and the meditation block, our subjects had to sit for 20 minutes, which were decomposed into 2 sub-blocks of 10 minutes each. The tradition-specific meditation corresponded to the second sub-block of the meditation block. To account for any potential effects on physiological measures of simply sitting still for a specific duration of time, we compared the 10-minute tradition-specific meditation block to the second block of the instructed mind wandering period (see Fig 2).

Robust linear correlations were computed using the skipped Pearson method implemented in the Robust Correlation Matlab toolbox from Pernet et al. [51]. This method protects against any bivariate outliers and uses 95% bootstrapped confidence intervals (CI) to assess significance. The R’s *ggplot2* package [52] was used to plot the distribution of data.

Finally, comparisons between groups in the psychometric and demographic sections were performed using Wilcoxon rank-sum test. Comparisons of a given measure within a group were performed using Wilcoxon signed-rank test.

## Results

### Demographic and psychometric data

Table 1 shows characteristics of each groups. There was no significant difference in age between groups (Wilcoxon rank-sum test, p>0.1). Regarding meditation experience, both the Vipassana and Himalayan yoga group spent significantly more hours in meditation than the Isha shoonya group (Wilcoxon rank-sum test, p<0.005). There was no significant difference in meditation experience between the Himalayan yoga and Vipassana group.

#### Meditation depth and thought density rating

Table 2 shows for each group and condition the mean scores to the meditation depth and thought density scales and the mean scores to the MEDEQ items “relaxation” and “hindrance”. Meditators successfully entered a meditative state during the experiment as assessed by the subjective reports that were given immediately after the meditation block. On a 0–10 scale (where 10 indicates the deepest meditative state they have experienced and 0 not deep at all) following the meditation period, the Himalayan yoga group reported an average meditative depth of 6.3 ± 1.8 (habitual meditative state rated 6.9 ± 1.3), the Isha shoonya group 7.5 ± 1.1 (habitual meditative state rated 7.0 ± 0.9), and the Vipassana group 6.0 ± 1.8 (habitual meditative state rated 5.1 ± 1.8). Note that the control group could not answer these questions since they did not have meditation practice to refer to. No significant differences were found between reported depth of meditation (during the experiment) relative to the reported depth of habitual meditation with the exception of a trend for the Isha shoonya group towards experiencing a deeper state of meditation than usual during the experiment (Wilcoxon signed-rank test, p = 0.05). Both the Isha shoonya and the Himalayan yoga groups reported a significantly deeper habitual state of meditation than the Vipassana group (Wilcoxon rank-sum test, p<0.01) with no significant difference found between the Isha shoonya and Himalayan yoga group in the ratings of their habitual depth of meditation (Wilcoxon rank-sum test, p = 0.6). After the meditation period, the reported depth of meditation was significantly higher in the Isha shoonya group as compared to the Vipassana group (Wilcoxon rank-sum test, p<0.01). No other significant differences were found between the groups on this measure. Ratings of thought density were significantly lower for all three groups after the meditation period than after the instructed mind-wandering period (HYT: mean score after meditation 4.5 ± 2.3, mean score after IMW: 6.6 ± 2.1, p = 0.01; ISY mean score after meditation 4.3 ± 1.4, mean score after IMW: 7.2 ± 2.3, p<0.01; VIP: mean score after meditation 4.1 ± 2.0 mean score after IMW: 6.0 ± 2, p = 0.01). No significant difference were found within the control group between ratings of thought density after the meditation period as compared to the instructed mind-wandering period (mean score after meditation 4.7 ± 2.0, mean score after IMW: 6.2 ± 2.3).

**Table 2:** Mean ratings on subjective reports and score on relaxation and hindrance MEDEQ items after meditation. “Habitual” stands for the experience participants usually have when meditating at home and “Exp.” stands for their experience during the experiment.

Groups	Meditation Depth		Thought Density		Relaxation		Hindrance	
	Habitual	Exp.	Habitual	Exp.	Habitual	Exp.	Habitual	Exp.
CTR	NA	NA	4.7 ± 2	6.2 ± 2.3	10 ± 3.8	10 ± 3.4	7.8 ± 5.1	7.7 ± 5.0
HYT	6.9 ± 1.3	6.3 ± 1.8	4.5 ± 2.3	6.6 ± 2.1	10 ± 4.8	11 ± 4.3	13 ± 5.1	10 ± 5.8
ISY	7 ± 0.9	7.5 ± 1.0	4.3 ± 1.4	7.2 ± 2.3	13 ± 3.0	11 ± 4.7	4.2 ± 4.5	9.0 ± 6.2
VIP	5.1 ± 1.8	6.0 ± 1.8	4.1 ± 2.0	6.0 ± 2	12 ± 3.4	10 ± 4.1	5.4 ± 4.7	9 ± 6.3

#### Relaxation and hindrance scores

Within group comparisons of scores on relaxation items after the meditation and instructed mind-wandering periods show that the Isha shoonya and Vipassana groups have significantly higher scores after the meditation period as compared to instructed mind-wandering period (ISY: p<0.0005; VIP: p<0.001, Wilcoxon signed-rank test, see Table 2). The Himalayan yoga and control groups showed no significant differences on these items between the meditation and instructed mind-wandering periods.

After the meditation period, both the Isha shoonya and Vipassana groups scored higher on the relaxation items than the controls and Himalayan yoga groups (Wilcoxon rank-sum test p<0.0005 for both Isha shoonya and Vipassana compared to control group; Vipassana compared to Himalayan yoga: p<0.01; Isha shoonya compared to Himalayan yoga: p<0.0001). The difference of score after the meditation period was significantly higher in the Isha shoonya group compared to the Vipassana group (p = 0.04).

Within group comparisons of scores to hindrance items after the meditation and instructed mind-wandering periods show that, for both Vipassana and Isha shoonya groups, scores were significantly lower after meditation than after instructed mind-wandering (VIP: p<0.0001; ISY: p<0.0001, Wilcoxon signed-rank test). Scores of the Himalayan yoga group were significantly higher after meditation than after instructed mind-wandering (p<0.01, Wilcoxon signed-rank test).

There were no significant difference in hindrance item scores after meditation and instructed mind-wandering session for the control group.

### Spectral analysis

**Fig 3:**
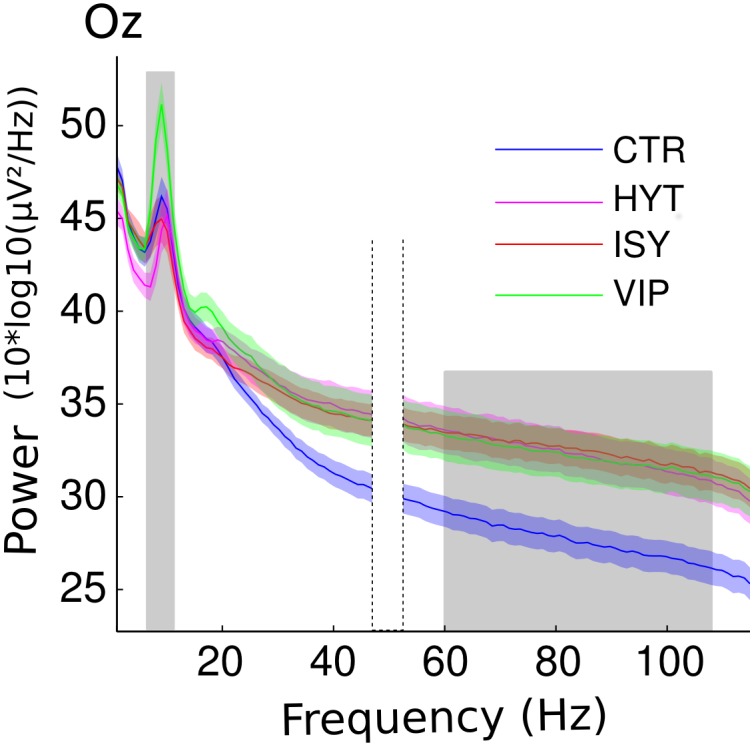
Spectral decomposition for occipital electrode Oz during the meditation condition in all 4 groups of subjects (CTR is the Control group; HYT is the Himalayan Yoga Tradition group; ISY is the Isha Shoonya Yoga group; VIP is the Vipassana group). Shaded regions indicate the standard error of the mean. The grey shaded area indicates the region of statistical differences between the groups after correction for multiple comparisons (see Material and Methods). The region around 50 Hz has been blanked out on the plot as it corresponds to line noise and has been excluded from analysis.

Frequency ranges of interest were defined by analyzing the spectral power from 2 to 110 Hz. Significant differences were observed between groups during the meditation period in the EEG spectral power, namely in the 60–110 Hz gamma frequency range and in the 7–11 Hz alpha frequency range (p<0.05, non-paired permutation t-test corrected for multiple comparisons—a representative electrode is shown on Fig 3).

No significant difference within groups was found for comparison between the meditation and instructed mind-wandering periods.

#### Gamma frequency band activity

**Fig 4:**
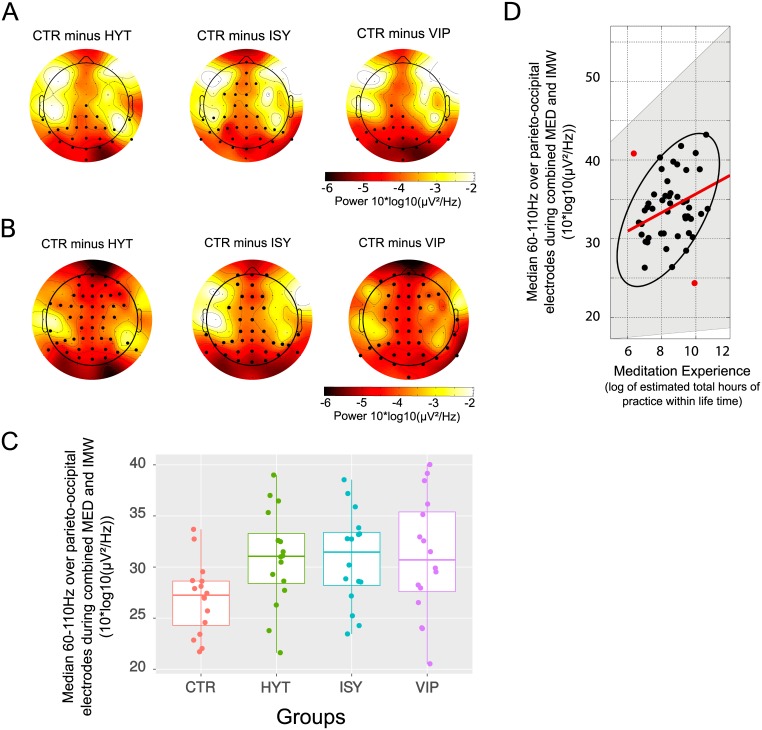
A. Spectral differences in the 60–110 Hz range between the control group and each of the meditator groups with MED and IMW conditions combined. A black dot at a given electrode site indicates significance at p<0.05 corrected for multiple comparisons (see Method). B. Spectral differences in the 60–110 Hz range between the Control group and each of the meditator groups during MED condition only. A black dot at a given electrode site indicates significance at p<0.05 corrected for multiple comparisons (see Method). C. Distribution of individual median 60–110 Hz activity over parieto-occipital electrode for combined meditation and IMW conditions in each group. D Median gamma power over parieto-occipital electrodes for the combined meditation and instructed mind-wandering conditions in the three meditator groups, as a function of log-scaled estimated hours of lifelong meditation practice (skipped Pearson’s r = 0.33, confidence interval (CI) = [0.06 0.53]). The grey shaded area shows the 95% bootstrapped CIs. A red dot denotes an outlier that was left out from the computation of the robust correlation analysis whereas the ellipse surrounds the data points included in it.

To assess the presence of a meditation trait effect, we first combined the two conditions meditation and instructed mind-wandering and took the median 60–110 Hz gamma power over the frontal and parieto-occipital electrodes. We then performed two one-way ANOVAs of the gamma power by group effect for each location (frontal or parieto-occipital) and found a significant effect of the group for the gamma power over parieto-occipital electrodes location (F(3,33) = 3, p = 0.03, explanatory measure of effect size = 0.39) but not for the gamma power over the frontal location (F(3,33) = 1, p = 0.36). We ran post-hoc tests over all electrodes within EEGLAB to compare each meditators group to the control group. Increased 60–110 Hz power over parieto-occipital electrodes was found in each group of meditators when compared to controls and additional increase over central and frontal electrodes was found for the Isha shoonya group only (non-paired permutation t-tests corrected for multiple comparisons, p<0.05. See Fig 4A). Fig 4C shows the individual distribution of the 60–110 Hz power over the parieto-occipital electrodes for the combined meditation and instructed mind-wandering conditions. We can see that despite a large individual variability within groups, the median gamma power in the three meditators groups is higher than the gamma power in most of the control subjects.

Next, we questionned more specifically the presence of a state effect by investigating the group and condition effects within the gamma range over parieto-occipital or frontal electrodes. For each case, the 60–110 Hz median gamma power for each four groups and each of the two conditions meditation and instructed mind-wandering was entered into an ANOVA mixed-effects model. We found a between-group effect on the median gamma in parieto-occipital electrodes location (F(3,60) = 3, p = 0.02) but not for frontal location (F(3,60) = 1, p = 0.1). The condition had a marginal effect when considering gamma power over posterior electrodes (F(1,60) = 3, p = 0.07) and no effect when considering frontal electrodes (F(1,60) = 0.6, p = 0.41). There was no group-by-condition interaction effect (F(3,60) = 0.7, p = 0.52 for posterior electrodes and F(3,60) = 2.1, p = 0.11 for frontal electrodes). Post-hoc tests over all electrodes location were run in EEGLAB to compare each group of meditators to the control group in each of the two conditions. When considering the meditation condition across groups, we observed higher 60–110 Hz gamma power in all of the meditator groups as compared to the control group over the frontal, midline and occipital electrode sites (non-paired permutation t-test corrected for multiple comparisons, p<0.05—see Fig 4B). When considering the instructed mind-wandering task, we observed a trend for increased gamma over occipital electrodes when comparing each of the three meditators groups to the control group (non-paired permutation t-tests corrected for multiple comparisons, HYT: p<0.06, VIP and ISY: p<0.08).

Specific effects of the different meditation practices were investigated by comparing the first 10 minutes of the meditation block in which participants practiced focusing on the breath with the last 10 minutes of the meditation block—corresponding to each group distinct meditation practice—taking only data from the meditator groups. We performed a mixed effects ANOVA taking the median gamma power during the meditation and breath focus conditions for the three meditator groups, leading to significant effect of condition (F(1,45) = 7, p = 0.01). However, the group effect and the group by condition interaction was not significant (respectively F(2,45) = 0.05, p = 0.9 and F(2,45) = 0.8, p = 0.4). Post-hoc tests showed that both Himalayan yoga and Isha shoonya showed decreased 60–110 Hz power in meditation compared to breath focus. This effect was observed over centro-occipital electrodes for the Himalayan yoga and over fronto-central electrodes for the Isha shoonya group (p<0.05, permutation of paired t-test corrected for multiple comparisons). The Vipassana group did not show any significant difference between breath focus and meditation in this frequency range.

The finding of significantly higher gamma power in meditators than in controls in combined meditation and instructed mind-wandering condition, and of a trend in the same direction when considering instructed mind-wandering alone supports the hypothesis of a trait effect of meditation practice on 60–110 Hz gamma power.

Following the finding by Lutz et al. [16] of a meditation state effect on the higher over lower frequencies ratio, we computed the ratio of spectral power between the 60–110 Hz range and the 2–11 Hz range for each group. No significant differences were observed between the meditation and instructed mind-wandering condition in any of the groups. Inter-group comparisons revealed a significantly higher 60–100 Hz over 2–11 Hz power ratio for Himalayan yoga group compared to control group only, both during meditation (p<0.005) and during instructed mind-wandering (p<0.05; two-tailed student t-test with 15 degrees of freedom corrected for multiple comparisons using Bonferroni approach). The Isha shoonya and Vipassana groups showed no significant differences from the control group with regards to the 60–100 Hz over 2–11 Hz power ratio and there was no significant difference either when comparing each group of meditator in a pairwise fashion.

#### Analysis of muscular and ocular artifactual activity

Research on the 30–90 Hz frequency range has previously linked gamma activity to various cognitive phenomena. However, recent research show that there may be contamination in these frequency bands due to eye [53] temporal, facial, and scalp muscle activity [54]. Activation of these muscles could result in significant increases in the power of high frequency bands and could potentially mask or bias the observed effects we report. In order to assess whether the increased power in the gamma band we observed in meditators was due to muscle or eye activity, we used the ICA algorithm to isolate and remove these artifacts [45]. To minimize the effects of subjective judgement, we only used components which represented unequivocal muscle activity based on their topographic and spectral characteristics.

**Fig 5:**
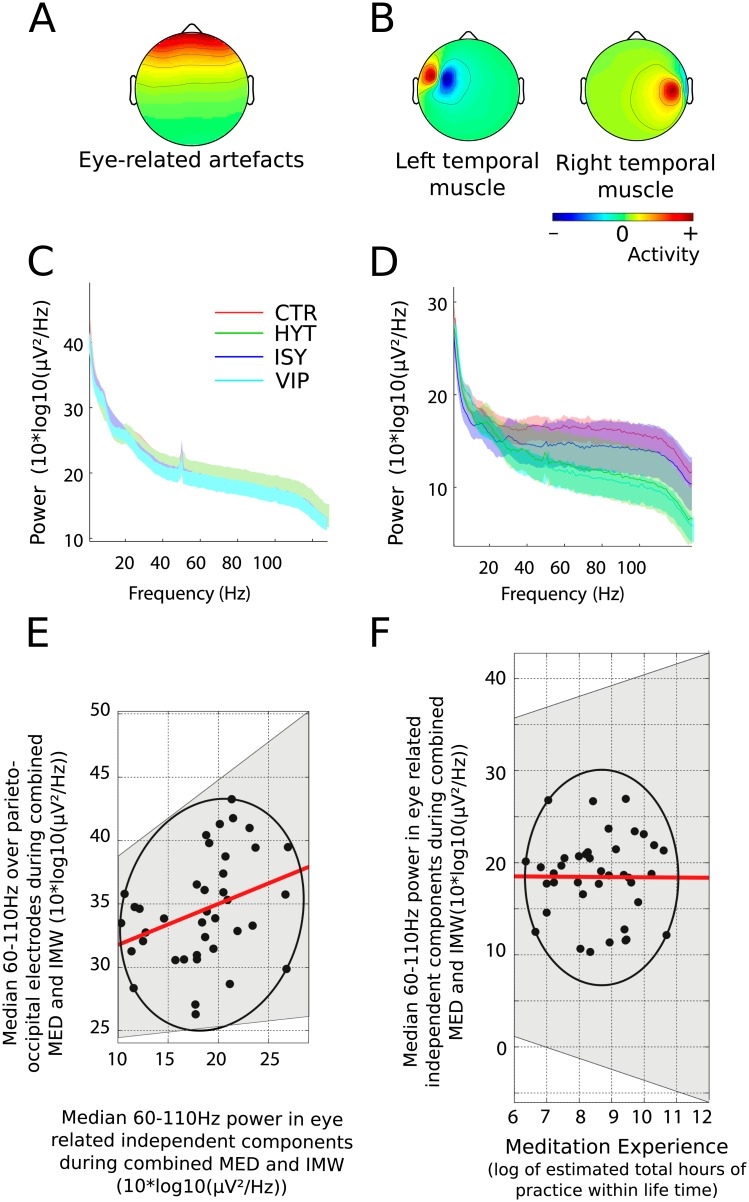
A. Average scalp topography of independent components’ activity corresponding to eye related artifacts. B. Average scalp topographies of independent components’ activity for left and right temporal muscle activity. C. No difference was observed between groups for eye movement independent components’ spectral activity in the meditation condition. D. Pooled left and right muscle ICA components spectral activity for the meditation condition indicate no significant difference between the 4 groups of subjects. E. There is a positive correlation between the 60–110 Hz activity in the components of eye related artifacts and the 60–110 Hz activity recorded over the parieto-occipital electrodes in combined MED and IMW conditions (skipped Pearson’ s *r* = 0.33, CI = [0.10 0.56]). F. The 60–110 Hz activity in the artifactual components related to eye artifacts do not correlate with meditation experience (skipped Pearson’s *r* = −0.006, CI = [-0.29 0.30]). In both panels C and D the shaded area around the curves represents the standard error to the mean. In panels E and F the shaded area represents the 95% confidence interval (CI).

**Fig 6:**
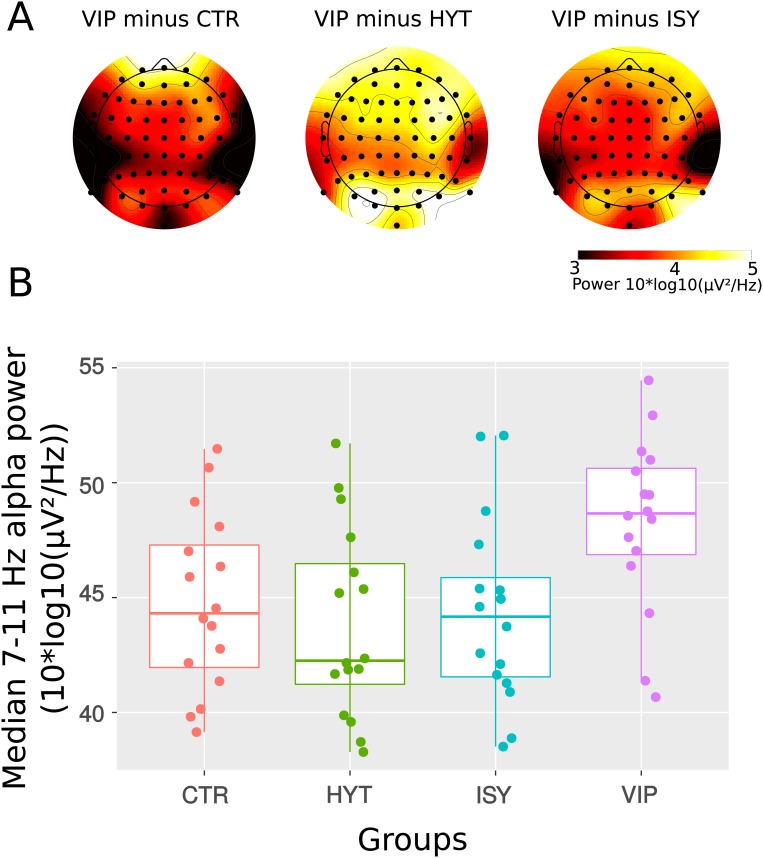
A. Spectral differences in the 7–11 Hz range between the Vipassana group and the control, the Himalayan Yoga and the Isha Yoga groups. A black dot indicates significance at p<0.01 for a given electrode site after correction for multiple comparisons (see Material and Methods). B. Distribution of individual median 7–11 Hz activity over all electrodes during meditation in each group.

Temporal muscle ICA components were identified by visual inspection of experimenter CB in all 64 subjects. Between 0 and 2 components were labeled as artifactual for each subject. Of these, 30 could be later aggregated at the group level in a cluster (see Methods). Within this cluster, 18 independent components corresponded to the right temple muscles (4 subjects in CTR group, 8 subjects in VIP, 3 subjects in HYT and 3 subjects in ISY) and 12 to the left temple muscles (2 subjects in CTR group, 2 subjects in VIP, 3 subjects in HYT and 5 subjects in ISY) (Fig 5B). These muscle artifacts had a spectrum dominated by high frequency activity over temporal electrode sites. No significant group or condition effect was found in the activity of the temporal muscles artifact components. If muscle activity from the scalp were responsible for the difference in gamma activity we observed between meditators and control subjects, it should have been visible (and amplified) in these components. In addition, we ran a correlation between the median spectral activity of these independent components present in the cluster corresponding to temporal muscles and the median 60–110 Hz gamma activity during combined meditation and instructed mind-wandering for the 24 subjects presents in the cluster. No significant correlation was found between the 60–110 Hz power and the activity of the cluster of artifactual independent components (Skipped Pearson *r* = −0.12, CI = [-0.5 0.33]). Furthermore, for all subjects, muscle-related artifactual component activity was removed at the pre-processing stage, before computing the spectral transformation of Figs 3, 4 and 6.

Additionally, experimenter CB manually identified ICA components accounting for eye movements in the 64 subjects. Artifactual components corresponding to ocular activity were identified by their typical scalp topographies with high amplitude signal over frontal electrode sites with relatively smooth 1/f spectrum. At the group level, 53 of these components could be clustered together (14 of the 16 subjects in the control, Vipassana and Himalayan yoga group and 11 of the 16 subjects in the Isha shoonya group) (Fig 5A). No significant spectral frequency power difference was observed for this ICA cluster between the 4 groups. If eye-movement related artifacts were responsible for the increased gamma power observed between groups in this dataset, we should have observed higher gamma power in the eye movement independent components for meditators compared to the control group. As for the muscle-related artifactual components’ activity, eye movement-related artifactual components’ activity was removed from the data at the pre-processing stage.

The analysis of eye-movement related artifacts activity between the meditation and instructed mind-wandering conditions shows that within the control group only, the spectral frequency power of the related ICA cluster is higher during instructed mind-wandering than during meditation in the range from 25 to 110 Hz (p<0.001). Within the same eye-movement related ICA cluster, we observed a trend for higher power in the 11–17 Hz frequency range during instructed mind-wandering compared to meditation in the Vipassana group (p<0.06) but failed to find any significant difference or trend in any frequency range when comparing meditation to instructed mind-wandering in the Himalayan yoga and Isha shoonya groups.

We observed a positive correlation between the activity of the cluster of IC corresponding to eye blinks and the activity of the 60–110 Hz frequency range power over parieto-occipital electrodes for datasets of the meditators presents in the IC cluster, in the combined meditation and instructed mind-wandering conditions (Pearson skipped r = 0.33, CI = [0.10 0.56]). However, the activity of this cluster of artifactual ICs did not correlate with the meditation experience of the corresponding meditator participants (Pearson skipped r = -0.006, CI = [-0.29 0.30]).

These results show that it is unlikely that the gamma power increase we observe in meditators in comparison to control subjects is due to either temporal muscles or eye artifacts. In addition, the scalp topography of the difference in EEG activity (Fig 4) is greater in medial frontal and occipital areas when compared to temporal areas, further indicating that classic temporal muscles related artifacts and lateral frontal artifacts are not likely to be contributing to the effects we are reporting in the EEG gamma 60–110 Hz frequency range.

#### Gamma activity correlation with meditation experience

Given that increased gamma power was observed for meditators relative to the control (see Fig 4), we investigated whether 60–110 Hz gamma power was positively correlated with the meditation experience of participants. To test this hypothesis, we extracted data from the three meditator groups corresponding to the combined meditation and instructed mind-wandering conditions for the parieto-occipital electrodes as they showed significant power spectrum difference with the control group (see Fig 4A). Hours of meditation experience were converted to a logarithmic scale to keep consistency with the expression of gamma power.

A positive correlation was found between the length of experience of meditation (expressed in log of hours) and the 60–110 Hz gamma power over parieto-occipital electrodes for the combined meditation and IMW conditions (see Fig 4D, skipped Pearson’s *r* = 0.33, CI = [0.06 0.53]).

In addition a positive correlation between gamma power over parieto-occipital electrodes and meditation experience was also found in the instructed mind-wandering only condition (skipped Pearson’s *r* = 0.31, CI = [0.01 0.55]). However, we failed to find a correlation with meditation experience when using the 60–110 Hz gamma power during meditation only (skipped Pearson’s *r* = 0.15, CI = [-0.12 0.42]).

This result of a positive correlation between gamma power in combined meditation and instructed mind-wandering conditions and the length of life time meditation practice further supports the hypothesis of a meditation trait effect within the EEG gamma frequency range.

#### Alpha frequency band activity

We performed a mixed-effects ANOVA to assess effects of groups and conditions in the alpha range (7 to 11 Hz). We found a significant effect of group (F(3,60) = 3, p = 0.02) but not of condition (F1,60) = 1.3, p = 0.2) and no significant group by condition interaction (F3,60) = 1, p = 0.3). Post-hoc between-group comparisons revealed that during the meditation period the 7–11 Hz power in the Vipassana group was greater than in the control group (non-paired permutation t-test, corrected for multiple comparisons, p<0.01), the Isha shoonya group (non-paired permutation t-test, corrected for multiple comparisons, p<0.01) and the Himalayan yoga group (non-paired permutation t-test, corrected for multiple comparisons, p<0.005) as shown on Fig 6A. These differences were significant at all electrode sites when comparing Vipassana to each of the other groups. Distribution of individual median 7–11 Hz over all electrode sites during meditation shows that except for three participants, meditators in the Vipassana group had higher 7–11 Hz power than most of the participants in the other groups (see Fig 6B).

During the instructed mind-wandering period, we observed higher 7–11 Hz power in the Vipassana group compared to the control group (non-paired permutation t-test, corrected for multiple comparisons, p<0.05, mainly at frontal and fronto-central electrodes sites), the Isha shoonya group (non-paired permutation t-test, corrected for multiple comparisons, p<0.05, over frontal and central electrodes sites) and the Himalayan yoga group (non-paired permutation t-test, corrected for multiple comparisons, p<0.05, at all electrodes sites).

These results show that Vipassana meditation is specifically associated with higher 7–11 Hz amplitude, both during meditation and instructed mind-wandering.

## Discussion

As summarized in Table 3, the main results of our study are twofold: first we demonstrated that when combining both the meditation and instructed mind-wandering periods, meditators from 3 different traditions exhibited higher gamma (60–110 Hz) spectral power over parieto-occipital electrodes as a trait effect when compared to control participants. When considering independently the meditation period, meditators from 3 different traditions relative to control participants showed higher 60–110 Hz spectral power mainly over occipital electrode sites but also, in a less robust way, over frontal and midline electrode sites. Of note, when considering instructed mind-wandering periods only, the increased gamma power in meditators compared to controls was observed as a trend. It is unlikely that the gamma effects were due to eye or scalp muscle artifacts as demonstrated by our analysis of independent components. In addition, we observed a positive linear correlation between the length of experience in meditation and the 60–110 Hz power over parieto-occipital electrodes in the combined meditation and instructed mind-wandering conditions. We also observed that Vipassana meditation practitioners exhibited higher power in 7–11 Hz alpha frequency band as a trait effect across both instructed mind-wandering and meditation conditions compared to other meditation groups and control participants.

**Table 3:** Summary of the main results.

	HYT	ISY	VIP
Gamma power in MED and IMW	higher 60–110 Hz power than CTR group in MED and MED+IMW; trend for higher 60–110 Hz power than CTR in IMW	higher 60–110 Hz power than CTR group,in MED and MED+IMW; trend for higher 60–110 Hz power than CTR in IMW	higher 60–110 Hz power than CTR group in MED and MED+IMW; trend for higher,60–110 Hz power than CTR in IMW
Gamma in breath focus	lower 60–110 Hz power in MED than in breath focus	lower 60–110 Hz power in MED than in breath focus	no significant difference between MED and breath focus
Alpha	-	-	higher 7–11 Hz power during MED and IMW than all other groups

### Psychometric data

Self-scoring after the meditation and instructed mind-wandering sessions suggests that all the meditation practitioners were able to enter a meditative state during the experiment. Ratings of meditation depth during the experiment did not significantly differ from ratings of habitual meditation depth, indicating that the experimental procedure still allowed subjects to reach their habitual meditation depth. In addition, ratings of thought density during the experiment were significantly lower for all groups of meditators after the meditation period than after IMW—a difference that was not found within the control group. Scores of the Vipassana and Isha shoonya groups were both lower on hindrance items and higher on relaxation items after the meditation session compared to the IMW session. Scoring of the control group on the relaxation and hindrance items did not differ between IMW and meditation.

However, the Himalayan yoga group might have experienced more difficulties than other meditator groups in establishing a meditative state within the laboratory environment: on the psychometric questionnaire, Himalayan yoga participants scores were higher on the hindrance items and lower on the relaxation items after the meditation than after the instructed mind-wandering session. Post-hoc analysis of the two specific hindrance items assessing drowsiness and mind-wandering supports this hypothesis: Himalayan yoga meditators scored higher on both these items after meditation than after instructed mind-wandering while within the meditation session they scored higher than all other groups of meditators and controls.

### Gamma activity as a trait effect of meditation

Our study demonstrates an increase in high-gamma 60–110 Hz associated with meditation expertise. High-gamma 60 to 110 Hz power effect was found during meditation in the three meditator groups compared to the control group but failed to reach significance when assessing the instructed mind-wandering state activity across or within groups. Specifically, comparisons between instructed mind-wandering and meditation periods within groups indicated trends but no specific state effects in the high gamma range for any of the groups. Comparisons between instructed mind-wandering periods across groups also showed a trend towards increased high gamma power over parieto-occipital electrodes in the meditator groups. The fact that these trends were observed, and that when grouping the meditation and instructed mind-wandering states we did observe a significant difference between meditators and controls, suggest that we are in the presence of a true trait effect.

Generally, our results are consistent with a limited number of previous studies demonstrating an enhanced 25–45 Hz gamma power in long-term practitioners. Higher occipital 35–45 Hz gamma activity was previously observed as a specific state effect in Vipassana meditators while meditating compared to a control condition of instructed mind-wandering similar to the one we used [17]. Another recent report showed that Vipassana/open awareness practitioners exhibited increased posterior 25–45 Hz gamma activity as both a trait and state effect of meditation [18]. Tibetan Buddhist meditators practicing non-referential compassion meditation also showed increased 25–42 Hz gamma power compared to control subjects as both a trait and state effect [16]. Increased gamma power (25–40 Hz) over parieto-occipital areas was also found in Tibetan Buddhist meditators as a meditation trait effect during NREM sleep [19]. Finally, both Ferrarelli et al. [19] and Hauswald et al. [20] report positive correlations between gamma power and the length of lifetime meditation, with Hauswald et al.’s results including the high gamma range (>60 Hz) [20].

As stated in the Introduction of this paper, there is as of yet no clear understanding of the functional role of EEG gamma activity in the literature. Physiological studies support the hypothesis of a role of 30 to 100 Hz gamma synchronization in facilitating the neuronal communication underlying conscious awareness in a meaningful way [22, 24]. Although frequently thought to be filtered out by skull and scalp tissues [55], high frequency gamma activity recorded on the scalp has been related to enhanced top-down control in vision [56], with strongest gamma power preceding fastest reaction time, and audition [57]. In addition, a magneto-encephalographic study reports a role of gamma >55 Hz in visual object coding (binding process) [58] and intracranial recording of EEG from the ventral occipito-temporal cortex also reported increased high frequency broadband gamma power related to selective attention [59].

In the light of this literature, we suggest that the trait parieto-occipital increase in high gamma we observe in meditators is a marker of an overall attentive state, the parietal cortex being associated with the focus of attention on a given object [60]. Furthermore, given the positive correlation observed between the length of lifetime practice of meditation and the high gamma power over parieto-occipital electrodes, we speculate that changes in meditators’ attention is due to neuroplasticity induced by their repeated practice of meditation.

Supporting this hypothesis we found a trait increase in the gamma over lower frequencies ratio, that is related to selective attention [61], when comparing the Himalayan yoga group to the control group both during meditation and instructed mind-wandering. This result was not found when comparing the Vipassana or the Isha yoga groups to the controls but no significant difference was observed either between the Himalayan yoga group and the these two groups, suggesting that the trait increase in gamma over lower frequencies ratio might still be present but to a lesser extent in Vipassana and Isha yoga practitioners.

Higher gamma synchronization in frontal areas was also found in all meditator groups relative to the control group during meditation. Of note, this effect observed over frontal electrodes observed only in post-hoc test may not be as robust as the effect observed over parieto-occipital electrodes. Increased synchronization of frontal high gamma activation has been reported in link with increased top-down control using intracranial EEG and magnetoencephalography [62, 63]. Phase coupling in the gamma range between frontal and parieto-occipital areas has also been linked with maintenance of visual images within working memory [64]. The gamma activity observed in the frontal areas of meditators relative to non-meditators might thus be related to more top-down control of attention, mediated by frontal cortical engagement and/or engagement of working memory processes. This top-down control may be exerted in order to redirect the attention towards the meditation task (for example in case of mind-wandering episodes) and to exert meta-cognitive processes in the observation of distracting internal (thoughts outside of present moment awareness) and external (discomfort, pain) stimuli. Working memory might also be particularly engaged in Himalayan yoga meditation which emphasizes to focus on the mental repetition of a mantra and on the breath and in Vipassana meditation which requires paying sequentially attention to somatosensory sensations on the body, while also holding attention on the breath. For Isha shoonya meditation however, it is unclear what type of object is fed to the attentional system since the explicit focus is on “nothingness”. Note that Isha meditators are the only group in which we found higher gamma power over frontal along with parieto-occipital electrodes as a trait effect and this might reflect different engagement of attentional processes in Isha meditation compared to Vipassana and Himalayan yoga meditation.

Finally, for the Himalayan yoga and Isha shoonya groups we observed increased high-gamma power during the breath focus period than during meditation—a difference that was not found within the Vipassana group. In Vipassana meditation, breath focus is a prominent part of the practice and it might have required more attentional effort for the Himalayan yoga and Isha shoonya group to practice awareness of the breath, which could explain that we found higher gamma power in breath focus than in meditation in these two groups.

### Gamma activity related to artifactual activity

Gamma activity in the 30 to 90 Hz range has been associated with eye tremors and microsaccades in addition to the well-known association with scalp and temporal muscles activity, thus rendering suspicious gamma findings in EEG studies [53, 65]. Gamma activity induced by microsaccades is most prominent when using the nose as an electrical reference whereas here we used the mastoid as a reference, which is less likely to be contaminated by microsaccades. In this report, we have also analyzed the ICA components associated with eyes and scalp muscles artifacts to assess the possibility that the observed gamma power differences may be spurious. No difference was observed between meditators and controls in the eye movement or temporal muscles independent components’ activity. It suggests that the higher gamma amplitude recorded in the meditator groups is most likely related to differences in cortical activation in the gamma frequency range. For the temporal muscles’ activity, no correlation was found with the activity within the gamma range of interest. However, we did find a positive correlation between the 60–110 Hz activity in the independent components corresponding to eye related activity and the gamma frequency range of interest. We think this has to be put in perspective with the fact that we did not find a correlation between this IC 60–110 Hz activity and the length of meditation experience, while we did find such a correlation when using the 60–110 Hz EEG activity. While it is possible that removal of the ICs corresponding to the eye related activity did not completely filtered out this artifactual activity from the EEG we believe that our signal remains mostly composed of genuine neuronal sources. As we have based our artifactual IC analysis using only ICs that unequivocally evoked muscle or ocular activity, we eliminated from the data ICs corresponding to specific groups of muscles. More work is needed to clearly identify ICs that would correspond to a state of general muscle tension so as to make sure the EEG is not contaminated at all by muscle artifacts, however this is beyond the scope of this article. We did not control for neck muscles activity and it could be argue that these muscles are responsible for the observed high gamma activity in meditators. Neck muscles activity is rarely removed from EEG data as it would require having specific electrodes on the neck area, and as otherwise it is not possible to isolate neck muscle artifacts. In addition, if we assume that neck muscles are responsible for the gamma effect we observe, we would expect that this activity would be larger for controls than for meditators. This is because meditators are able to remain perfectly immobile during the recording session, with minimal posture adjustment. A key principle of meditation is that stillness of the mind requires stillness of the body. By contrast control subjects might have had a harder time to maintain their posture, requiring more adjustment and therefore more muscle activity. However, we observed the opposite in our data with more high frequency activity being associated with meditation.

Another related possible confounding factor comes from the fact that, to perform the experiment, control participants sat on a chair more often and were generally adopting a less tonic posture than meditators. It is then possible that a tonic posture is possibly more arousing and therefore leading to enhanced EEG fast frequencies in meditators. In our study 14 of the controls sat on a chair as well as 2 of the Himalayan yoga, 8 of the Isha shoonya and 4 of the Vipassana participants. The Himalayan yoga group who sat the most on the ground does not show higher gamma power than the Isha shoonya group (in which half participants sat on a chair) or the Vipassana group (in which 1/4 of participants sat on a chair). While such posture effect should be more thoroughly investigated, our study does not support the hypothesis that the results we observed in the gamma band are primarily due to subjects’ posture.

### Alpha activity

We have found a trait related increase in amplitude in the 7–11 Hz alpha frequency band that was specific to the Vipassana group relative to all other meditator and control groups.

To our knowledge, there is no previous study showing increased alpha frequency amplitude in Vipassana meditators relative to meditators of other traditions (for a recent review see [66]). The litterature reviewed in [15] reports increased of alpha amplitude in a variety of meditation practices (ie. Transcendental Meditation, Zen, Sahaja Yoga, etc.) when compared to control states. However none of these studies included Vipassana meditators.

Research shows that increases in alpha power are generally not reliable markers of meditative states [17]. In line with previous findings [17], no significant differences were found between alpha amplitude during meditation and the control mind-wandering task in the Vipassana group. The observed state-related decrease in high alpha (10–11 Hz) power in the Himalayan yoga group is discussed further below.

Increases of alpha power have been observed in tasks requiring the redirection of attention towards internal objects—for example when subjects are asked to imagine a stimulus—supporting the hypothesis that alpha power inhibits irrelevant sensory inputs [67]. More recently, modulation of the alpha rhythm has been shown to play a part in selective attention processes by regulating thalamocortical sensory transmission [68] and thus participating in functional inhibition [69, 70].

A recent model proposes that through the training of localized mindful attention to somatic sensations, mindfulness meditators learn to control alpha oscillations so as to suppress irrelevant sensory input in a top-down fashion [71]. Thus, the global increase of alpha amplitude observed in Vipassana meditators only may be related to the specificity of a meditation style that starts with a somatically focused mindful attention before moving towards mindful open monitoring. Based on this model, we hypothesize that trait changes in alpha amplitude in Vipassana meditators may be due to their practice of embodied mindfulness which may increase internalized focus and increase gating of distracting stimuli. Inhibiting irrelevant sensory inputs might not be as important in the other assayed meditation traditions, where attending selectively to some types of endogenous sensory stimuli to the exclusion of others is not as central to the practice.

Our results show higher alpha power at all electrode sites in the Vipassana group compared to all other groups during meditation while during instructed mind-wandering, alpha power in the Vipassana group was greater at all electrode sites relative to Himalayan yoga but only at frontal and central sites relative to controls and Isha shoonya meditators. An hypothesis is that both Himalayan yoga and Vipassana practitioners who have significantly more hours of meditation experience than Isha shoonya practitioners and controls, display meditation trait effects in the alpha frequency range, with Vipassana practitioners having enhanced inhibitory mechanisms throughout the brain and Himalayan yoga practitioners having constantly less activation of these mechanisms (which may be due to trying to hold their mantra in their mind). By contrast, it is possible that during the instructed mind-wandering session control participants and Isha shoonya meditators used a higher degree of inhibitory mechanisms to focus on their thoughts, thus reaching a level of alpha activation equivalent to Vipassana.

### Meditation state and trait effects

No meditation state effects were found. As such, our study contrasts with other EEG studies finding state effects in the EEG power spectrum in various meditation practices [16–18, 72]. An explanation for this result might be important subject variability during the instructed mind-wandering task. Many other published studies used “rest” as a control state (see for example [16, 18, 36, 72, 73]). We attempted to use the instructed mind-wandering task as a control state in order to decrease variability during the control state but it is possible that this is not an effective method to do so. Our control task could have recruited similar brain mechanisms to monitor mental activity as did the meditation task. In fact, although spontaneous mind-wandering has been reported as linked to default-mode network (DMN) activation [74–77] while meditation is associated with DMN deactivation [78, 79], explicitely instructing participants to mind-wander through their autobiographical memory is likely to require conscious monitoring of one’s own mental activity, involving the same cognitive control networks as in meditation [78]. The difference might lie in the internal attitude subjects adopt towards thoughts: during meditation the participants are attempting to focus their awareness away from mind-wandering and autobiographical thoughts, towards the present moment awareness of the meditative object. During the instructed mind-wandering they are explicitly told to let their mind freely wander through their memories and autobiographical thoughts of the past. It is likely that during the instructed mind-wandering task some of our meditators kept a similar non judgmental attitude towards their experiences that they have when meditating. Another issue is that during meditation, even for experienced meditators, mind-wandering cannot be avoided. It is thus likely that some of our meditators had episodes of mind wandering during the meditation task and that some had episodes of meditation during the mind-wandering task.

However, we are reporting a meditation trait effect in the high gamma 60–110 Hz frequency range over the parieto-occipital electrodes in all the three types of meditation we studied here. In addition, a trait effect in the alpha 7–11 Hz range is observed over all the scalp electrodes in the Vipassana group.

In further support for a meditation trait effect on the gamma frequency band, comparison between the meditation and breath focus periods reveal that for the Vipassana group the global increase of high-gamma power is present also in the breath focus practice. However, in the Isha shoonya and Himalayan yoga groups there was a state effect with decreased gamma frequencies amplitude during meditation—suggesting that different processes are involved in Himalayan yoga and Isha shoonya groups in the practice of breath focus compared to their specific meditation styles, a difference that may be less present in the Vipassana group.

We thus observed more significant trait effects than state effects between the meditator groups when comparing the meditative and instructed mind-wandering states and when comparing the breath focus and meditation practices, at least for the Vipassana group. Showing that different meditation traditions share common neural correlates is important because it indicates that the clinical benefits of these practices [1, 80] could be similar. Further research will be necessary to investigate this hypothesis.

### Limitations of the study

Our study involved self-selection of the participants and only collected data at one point in time. Longitudinal studies of meditative practice with random selection of the participants training in meditation would be useful in the delineation of the likely complicated interplay of neurophysiological state and trait effects as they sequentially engage over the course of meditative training. Such studies could also help unravel individual differences in the pre-meditation training baseline that can influence the development of trait and state effects of meditation. One could also argue that our results might have been influenced by the fact that during the meditation condition, the control participants did 2 times 10 minutes of the same breath focus task whereas the meditators did 10 minutes of breath focus and then 10 minutes of a new task in form of their specific meditation practice. However, as breath focus is part of the meditation training for all three groups of meditators we do not think the switch towards performing the actual meditation practice could have been seen as starting a new fresh task that could have biased our results.

## Conclusion

We have provided evidence that daily meditation practice is correlated to both state and trait changes in the observed amplitude of brain electrical oscillations of three different meditation practices. These changes do appear to vary across meditative techniques but one shared feature appears to be enhanced gamma power in the parieto-occipital area. In addition, one specific finding that seems to be unique amongst these three groups of meditative practice is the enhanced alpha power seen as a trait effect in Vipassana practitioners relative to control subjects, Isha shoonya yoga and Himalayan yoga tradition practitioners. Further EEG studies of meditation should favour comparative designs to help move forward our understanding of the neuronal basis of meditation practice.
